# Potential Tumor Suppressor Role for the c-Myb Oncogene in Luminal Breast Cancer

**DOI:** 10.1371/journal.pone.0013073

**Published:** 2010-10-07

**Authors:** Aaron R. Thorner, Joel S. Parker, Katherine A. Hoadley, Charles M. Perou

**Affiliations:** 1 Curriculum in Genetics and Molecular Biology, University of North Carolina, Chapel Hill, North Carolina, United States of America; 2 Department of Genetics, University of North Carolina, Chapel Hill, North Carolina, United States of America; 3 Lineberger Comprehensive Cancer Center, University of North Carolina, Chapel Hill, North Carolina, United States of America; 4 Department of Pathology and Laboratory Medicine, University of North Carolina, Chapel Hill, North Carolina, United States of America; Roswell Park Cancer Institute, United States of America

## Abstract

**Background:**

The transcription factor *c-Myb* has been well characterized as an oncogene in several human tumor types, and its expression in the hematopoietic stem/progenitor cell population is essential for proper hematopoiesis. However, the role of *c-Myb* in mammopoeisis and breast tumorigenesis is poorly understood, despite its high expression in the majority of breast cancer cases (60–80%).

**Methodology/Principal Findings:**

We find that *c-Myb* high expression in human breast tumors correlates with the luminal/ER+ phenotype and a good prognosis. Stable RNAi knock-down of endogenous c-Myb in the MCF7 luminal breast tumor cell line increased tumorigenesis both *in vitro* and *in vivo*, suggesting a possible tumor suppressor role in luminal breast cancer. We created a mammary-derived *c-Myb* expression signature, comprised of both direct and indirect c-Myb target genes, and found it to be highly correlated with a published mature luminal mammary cell signature and least correlated with a mammary stem/progenitor lineage gene signature.

**Conclusions/Significance:**

These data describe, for the first time, a possible tumor suppressor role for the *c-Myb* proto-oncogene in breast cancer that has implications for the understanding of luminal tumorigenesis and for guiding treatment.

## Introduction

Breast cancer is a heterogeneous disease and numerous studies have defined at least five molecular subtypes of breast tumors using an “intrinsic” gene set [1], [2], [3], [4]. The luminal/estrogen receptor-alpha positive (ER+) subtypes are the most commonly diagnosed breast cancers (60–80%), with patients being classified as either good outcome Luminal A, or worse outcome Luminal B. Patients with Luminal A tumors have good overall survival, in part, because these tumors are slow growing, typically respond to endocrine therapy, and have infrequent *TP53* mutations [5], [6]. The luminal subtypes are defined by high expression of approximately 80 genes within the intrinsic classification gene list including *ESR1*, *GATA3*, *FOXA1*, and *c-Myb*, the latter of which is a previously described proto-oncogene frequently observed as amplified in a variety of tumor types [7].

Nearly three decades ago the *c-Myb* transcription factor was identified as the mammalian homolog of *v-myb*, a transforming retroviral oncogene linked to avian leukemia [7], [8], [9]. Since that time, *c-Myb* high expression has been associated with oncogenic activity and poor prognosis in several human cancers including T-cell leukemia, acute myelogenous leukemia, colorectal tumors, and most recently in adenoid cystic carcinomas [10], [11]. In addition, *c-Myb* has been implicated in progenitor cell maintenance and is required for proper cellular differentiation in the hematopoietic system, neuronal cells, skin cells, and colonic crypts [12], [13], [14], [15]. *c-Myb* high expression is frequently associated with a variety of immature cell lineages, and expression levels decrease as cells differentiate [16]. However, there is little known about the role of *c-Myb* in normal mammopoeisis and breast tumorigenesis, despite its high expression in virtually all ER+ tumors as well as in 29% of hereditary (typically ER-negative) BRCA1 breast cancers [17], [18].

To gain insight into *c-Myb* and its involvement in breast cancer, we analyzed the expression of *c-Myb* in the context of breast tumor subtypes, and examined its association with patient outcomes. We also manipulated the c-Myb protein levels via RNA interference in a Luminal/ER+ mammary cell line, observed alterations in growth properties both *in vitro* and *in vivo*, and identified a mammary-specific *c-Myb* gene signature.

## Results

### *c-Myb* high expression correlates with luminal subtype

**Figure 1:**
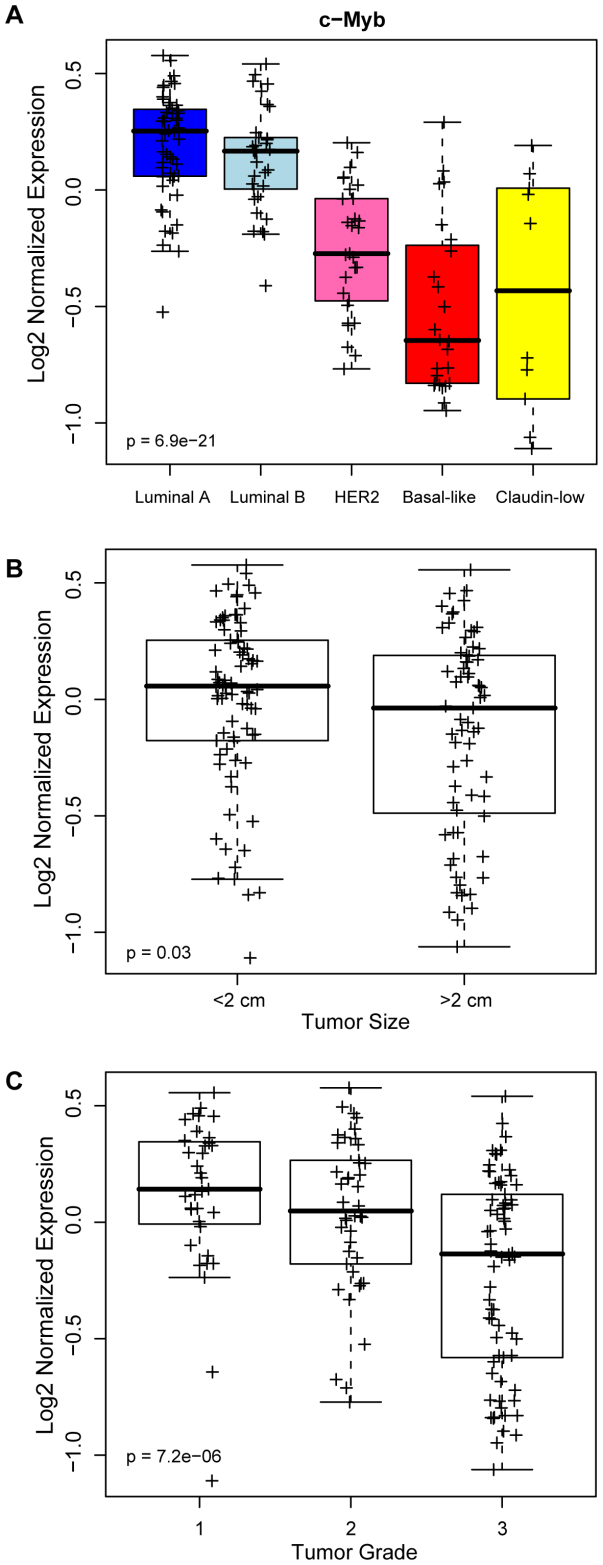
*c-Myb* high expression correlates with luminal subtype, small tumor size and low tumor grade breast cancers. (A) *c-Myb* expression varied across breast tumor subtypes in the NKI local-only treated tumor dataset (n = 147). The relationship of *c-Myb* gene expression to (B) tumor size and (C) tumor grade was also tested. Statistical significance determined by t-test or ANOVA.

To study the role of *c-Myb* in breast tumors, we first examined associations between *c-Myb* mRNA expression and tumor subtype. Gene expression profiles of locally-treated (no adjuvant systemic therapy) breast tumors from the Netherlands Cancer Institute microarray dataset (local-only Tx: NKI-147) [19] were classified into the intrinsic subtypes (Luminal A, Luminal B, HER2-enriched, Basal-like, and Claudin-low) using the PAM50 and Claudin-low classifiers, as described in [3], [4]. An ANOVA was performed to determine statistical significance of *c-Myb* expression across the breast cancer subtypes (Fig. 1A). Expression of *c-Myb* differed significantly across the subtypes with highest expression observed in the ER+ Luminal A and B subtypes and lowest expression in the Basal-like/ER- tumors. Luminal *c-Myb* expression versus non-Luminal was also significant (p = 0.001). High *c-Myb* expression levels also significantly correlated with a smaller tumor size and lower grade (Figs. 1B and C). Similar results were observed using two other breast tumor microarray datasets, including a University of North Carolina dataset (GSE18229) that includes normal mammary tissue and the Miller *et al*., 2005 (GSE3494) dataset consisting of primary invasive breast tumors (Fig. S1A and data not shown) [4], [20].

### *c-Myb* high expression correlates with good prognosis

**Figure 2:**
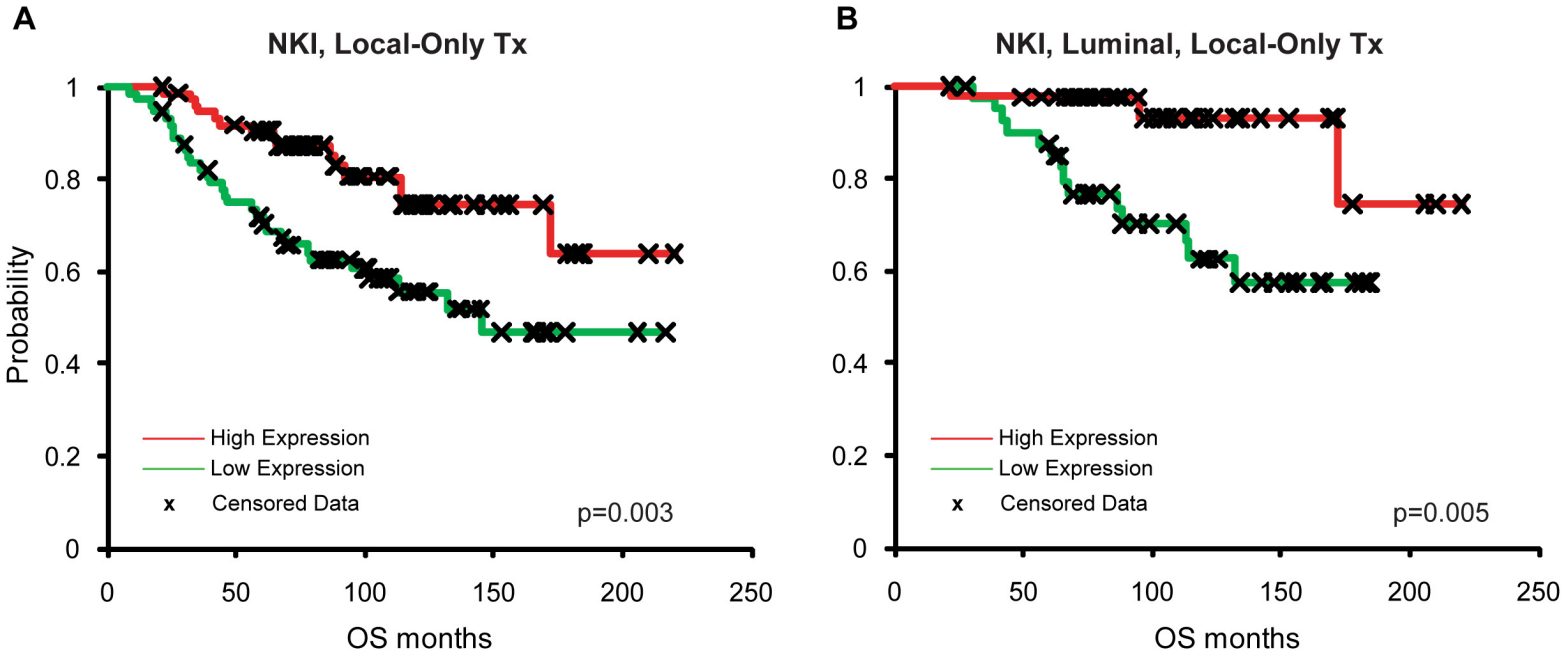
*c-Myb* high expression correlates with good prognosis across all breast cancer patients and within luminal tumors. Kaplan-Meier overall survival (OS) analysis based on *c-Myb* expression values rank ordered (high-to-low) and divided into halves. NKI-147 (A) All patients (n = 147), (B) Luminal A and B subtypes only (n = 84).

Next, we examined associations between *c-*Myb mRNA expression and patient outcomes. Using this same NKI-147 dataset, tumors were rank ordered and separated into high and low groups based on *c-Myb* mRNA expression levels and were analyzed for overall survival (OS) using Kaplan-Meier analysis of all tumors, as well as within each subtype. High *c-Myb* expression levels significantly correlated with better survival across all subtypes (Fig. 2A, n = 147), as well as in the combined Luminal A+B subtypes (Fig. 2B, n = 84) and the Basal-like subtype (Fig. S2A; n = 24), but not in the HER2-enriched or Claudin-low subtypes (Figs. S2B, n = 29; S2C, n = 10).

We also investigated if *c-Myb* expression levels correlated with achieving a pathological complete response (pCR), which is a measure of tumor response to neoadjuvant chemotherapy. Using published data [21] consisting of microarrays on breast tumors of patients receiving neoadjuvant paclitaxel, and subsequent 5FU-Adriamycin-Cyclophosphamide (T/FAC; n = 133), we observed that high *c-Myb* levels significantly correlated with a low pCR rate (p = 0.03; Table S1). This finding is consistent with previous findings that high ER levels predict low pCR rates [21], as does being of the Luminal A subtype [3].

### *c-Myb* knock-down in MCF7 cells increases tumorigenesis *in vitro* and *in vivo*

**Figure 3:**
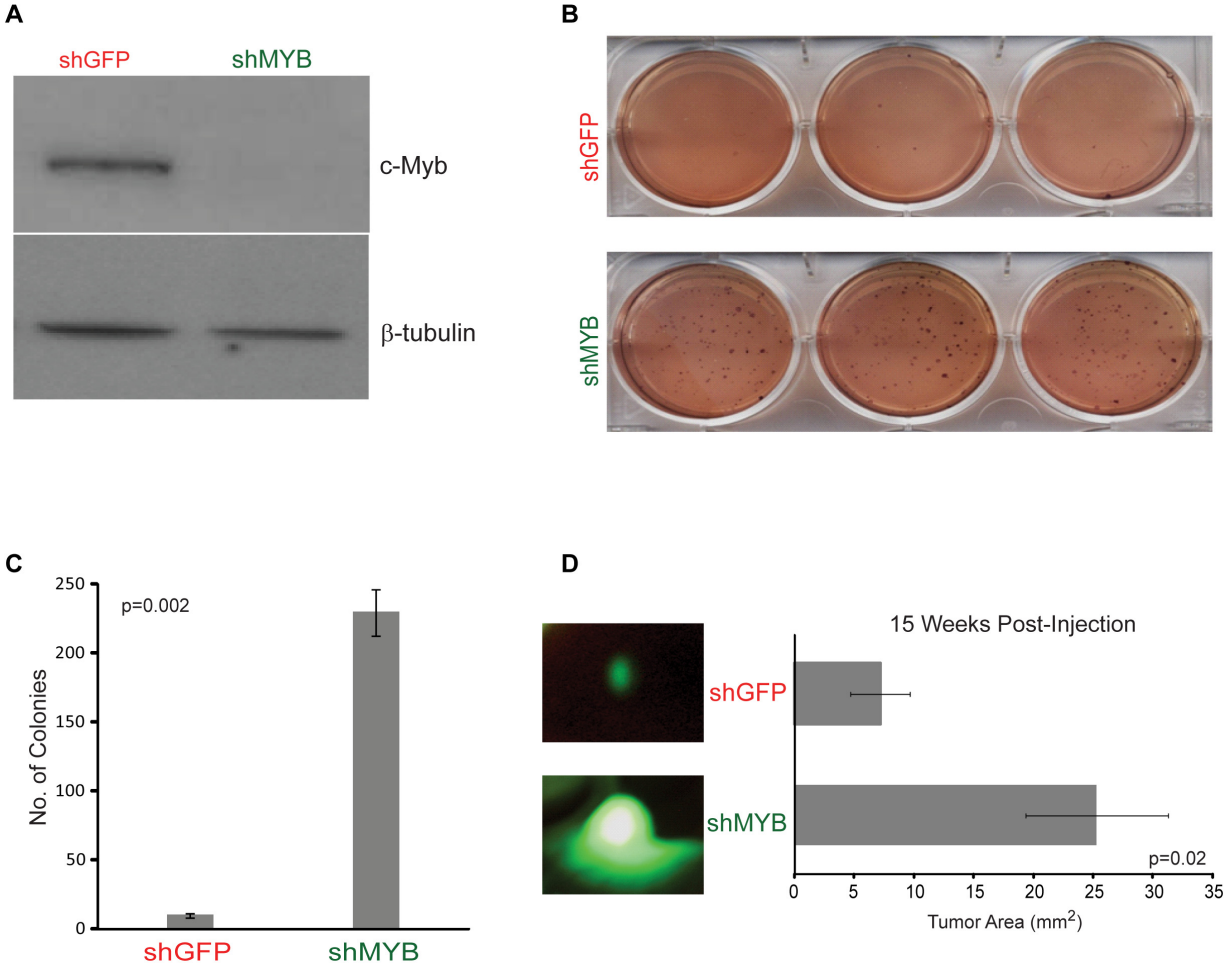
c-Myb knock-down increases luminal tumor growth both *in vitro* and *in vivo*. (A) Western blot analysis of c-Myb knock-down (shMYB) versus control (shGFP) in MCF7 cells. β-tubulin was used as a loading control. (B) Representative dishes displaying the effects of c-Myb knock-down in a soft agar colony formation assay. (C) Quantification of colony formation assay. Statistical significance determined by two-tailed independent t-test and error bars represent standard deviation. (D) Tumor area of nude mice fifteen weeks after injection of MCF7 shGFP or shMYB; representative images were taken of each tumor type in live mice. Error bars represent standard error and p-value calculated by a two-tailed independent t-test.

Based on our observations that higher levels of *c-Myb* are predictive of good outcomes in all breast tumors, as well as in luminal tumors, we utilized RNA interference (short hairpin RNA, shRNA) to knock-down endogenous c-Myb protein in the luminal tumor-derived cell line MCF7. Microarray analysis revealed that transcript levels of *c-Myb* were decreased 2.5-fold in the stable *c-Myb* knock-down (shMYB) relative to the control (shGFP; data not shown), while western blot analysis showed little to no detectable c-Myb protein expression in shMYB cells (Fig. 3A). A cell proliferation assay was performed to compare the doubling time of shMYB versus shGFP. shMYB cells grew faster *in vitro* (cell doubling time, hours: shGFP, 23.7±1.1; shMYB, 20.5±0.8) than empty vector controls.

Furthermore, both cell lines were tested for anchorage-independent growth by means of colony formation in soft agar. shMYB cells formed nearly 14-fold more colonies than shGFP (Figs. 3B and C). Next, we assayed both cell lines for their relative sensitivities to tamoxifen and found that shMYB cells were more resistant to tamoxifen based on IC50 estimates (Table 1).

**Table 1:** 72 h inhibitory concentration (IC50) for tamoxifen in MCF7 lines.

MCF7 Cell Line	IC50 (nM)	Standard Deviation
shGFP	6.5	6.2–6.9
shMYB	7.9	7.0–8.9

To examine if the *in vitro* data were relevant in *in vivo* xenografts, we stably expressed ZsGreen1, a reef coral fluorescent protein, in both of the MCF7 cell lines (shMYB and shGFP) to allow for ease of *in vivo* tumor visualization. Nude mice (without estrogen pellets) were injected with 5.5×10^5^ cells that were embedded in Matrigel into each fourth mammary gland (shGFP, n = 10; shMYB, n = 9). Fifteen weeks post-injection representative images were taken of tumors in live mice, and tumor area calculated. Tumors derived from the shMYB cell line were significantly larger than controls (Fig. 3D).

### *c-Myb* expression signature identifies many Luminal/ER+ subtype defining genes

In order to identify both direct and indirect transcriptional targets of c-Myb in breast cells, Agilent microarrays were used to assess the gene expression differences between shMYB cells (n = 5) versus shGFP cells (n = 6). In a two-class Significance Analysis of Microarrays (SAM) analysis [22], 2,088 significantly differentially expressed genes were identified using a false discovery rate (FDR) of less than 1%, hereafter termed the “c-Myb gene signature” (Table S2). Several previously identified c-Myb target genes were on this list including *KIT*, *DHRS2* (*Hep27*), and *EMP2* [23], [24], [25].

*c-Myb* is a possible estrogen receptor target gene [26], and is repeatedly observed as being highly expressed in the luminal “intrinsic” gene set [27], [28]. To determine if genes within the *c-Myb* gene signature overlap with the luminal intrinsic gene set, we used the published human breast tumor microarray data (n = 232) of Herschkowitz *et al*., 2007 [29], clustered the tumors using the ∼2,000 intrinsic gene list described by Parker *et al*., 2009, and defined the luminal cluster as genes highly correlated (0.65 node correlation; 79 genes total) with *ESR1*, a central gene in the luminal cluster (Fig. S3 and Table S3). We also analyzed previously published *ESR1* and *GATA3* gene signatures to determine their luminal cluster contributions [27], [28]. The *c-Myb* signature had the largest number of genes overlapping with the luminal cluster (24%), followed by *GATA3* (10%) and *ER* (4%) signature genes, and unique combinations of the signatures accounting for 6% (Table S3). These data suggest that the Luminal/ER+ cluster is a combination of the effects of multiple transcription factors, with c-Myb being the major contributor *in vivo* identified thus far.

### *c-Myb* gene signature correlates with mature luminal mammary cell lineage

**Figure 4:**
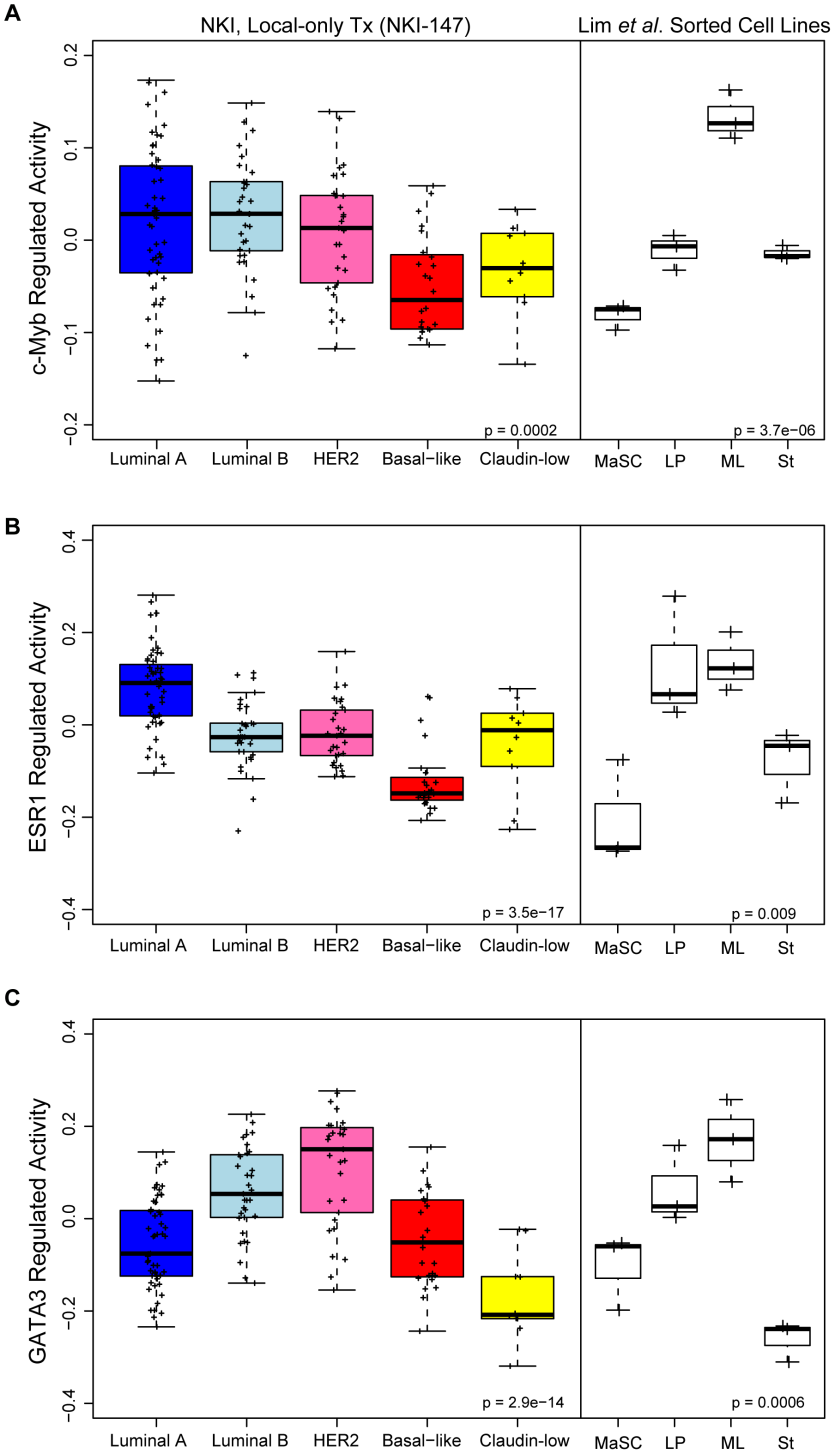
*c-Myb* gene signature correlates with mature (differentiated) luminal mammary cells and luminal tumors. (A) *c-Myb*, (B) *ESR1*, and (C) *GATA3* gene signatures across NKI-147 or mammary gland cell lineage gene signatures [30]. Tumor gene expression was weighted according to *c-Myb*, *ESR1*, or *GATA3* gene signatures by calculating the inner product of each signature SAM statistic and the tumor expression profile. Lim *et al*. lineage signatures were processed in the same fashion as tumors to demonstrate the developmental context of these signatures. MaSC: mammary stem cell-enriched; LP: luminal progenitor; ML: mature luminal; St: Stromal.

Recently, Lim *et al*. used florescence activated cell sorting (FACS) of normal, human mammary tissue to isolate four discrete cell populations within the mammary gland hierarchy (i.e. stromal, mammary stem cell, luminal progenitor, and mature luminal populations) and performed microarray analysis [30]. An “activation” or “regulated activity” status was ascertained for each of our cell line-derived *ESR1*, *GATA3*, and *c-Myb* gene signatures and applied to the Lim *et al*. the mammary gland lineage expression data (Figure 4, right). The differentiated, mature luminal lineage displayed high c-Myb regulated activity that was not observed in the other cell populations, including the mammary stem cell population (Fig. 4A). The *ESR1* gene signature was significantly correlated with both the luminal progenitor and mature luminal populations, whereas the *GATA3* gene signature was highly correlated with the mature luminal population, and to a lesser extent, the luminal progenitor lineage (Figs. 4B and 4C, respectively).

Tumor gene expression profiles (NKI-147) were processed in the same fashion as the sorted cell lines and tumor profiles were compared to the *ESR1*, *GATA3*, and *c-Myb* gene signatures to demonstrate which breast tumor subtypes were enriched for these signatures (Fig. 4, left). The *c-Myb* gene signature was strongly correlated with the Luminal A, Luminal B, and HER2-enriched subtypes, and was least correlated with Basal-like tumors (Fig. 4A). The *ESR1* gene signature was highest in the Luminal A subtype and lowest in the ER-negative, Basal-like tumors (Fig. 4B). The *GATA3* gene signature was most strongly correlated with the HER2-enriched subtype (Fig. 4C). Similar results were observed when looking at the individual gene expression of *ESR1*, *GATA3*, or *c-Myb* across both the Lim *et al*. and tumor data (Fig. S4). The above analyses were repeated using a UNC breast tumor gene expression dataset that also included normal mammary tissue [4] (n = 324) and similar results were observed (Figs. S1 and S5).

## Discussion

The essential role of the *c-Myb* oncogene in leukemogenesis has been appreciated for several decades. Its expression is required for maintenance of both acute and chronic myeloid leukemia cells [31], [32]. *c-Myb* is also necessary for normal hematopoiesis; knock-out mice succumb to embryonic lethality (e15) due to unsuccessful blood cell lineage formation [15], and lineage-specific knockouts have revealed that *c-Myb* is required for proper T-cell differentiation [33]. Studies in colorectal carcinoma have found high *c-Myb* expression to correlate with poor prognosis [10], and more recently the fusion of *c-Myb* and the transcription factor *NFIB* has been reported as a potential “hallmark” of adenoid cystic carcinomas [11]. Based on these data it has been hypothesized that the *c-Myb* oncogene is necessary for progenitor cell maintenance, and decreasing c*-Myb* expression is crucial for cellular differentiation [7].

Our data in breast tumors, however, suggest a possible tumor suppressor role for *c-Myb*, where high expression portents a good outcome and high differentiation status of tumors. It is known that *c-Myb* expression can be modified by the estrogen receptor, and c-Myb protein levels are increased in ER+ tumors [18], [34], [35]. Indeed, twenty years ago Guerin and colleagues described a correlation linking high c-Myb levels with several good prognostic features, such as low tumor grade, ER+ status, and an anti-correlation to poor outcome inflammatory breast carcinomas [18]. Here, our findings confirm that *c-Myb* high expression correlates with the ER+ Luminal A and B subtypes of breast cancer as well as smaller tumor size and lower tumor grade (Fig. 1). Unlike the other tumor types discussed above, breast tumors having high *c-Myb* expression levels show a good prognosis, even within luminal breast tumors alone (Fig. 2). These data describe, for the first time, that *c-Myb* is a prognostic feature in breast cancer. We purposefully used a microarray dataset where patients received no adjuvant systemic therapy so as not to confound the survival data with the use of chemotherapeutics or estrogen receptor antagonists.

To analyze the role of *c-Myb* in breast tumorigenesis, we knocked-down endogenous c-Myb levels in MCF7 cells, a luminal and estrogen-responsive breast tumor cell line [36]. When c-Myb protein levels were significantly decreased relative to the parental cell line, we observed faster cell growth, increased tamoxifen resistance, and increased colony formation *in vitro* (Figs. 3B–C, Table 1). These observations were also preserved *in vivo*, where c-Myb-depleted MCF7 cells injected into nude mice grew significantly faster than MCF7 controls (Fig. 3D); many of the control tumors did not grow or disappeared by 15 weeks post-injection. This suggests that the c-Myb knock-down cell line is less estrogen-dependent than the parental line, given that no exogenous hormone was implanted in these mice. This result also correlates with the observed decreased tamoxifen sensitivity in the c-Myb knock-down cells.

A recent study found that the estrogen receptor can directly relieve transcriptional attenuation in the first intron of *c-Myb*, thus giving greater insight into how ESR1 regulates *c-Myb* expression [34]. These authors proposed that c-Myb is required for the proliferation of MCF7 cells because they observed decreased proliferation in c-Myb knock-down MCF7 cells versus controls when treated with β-estradiol. These results are contradictory to ours, where we observed increased growth and tumorigenesis in the c-Myb knock-down line. This may be in part due to our use of a more estrogen sensitive MCF7 cell line; unlike the cell line used by Drabsch *et al*., our MCF7 isolate requires estrogen to grow and in the absence of hormone (i.e. phenol-red free and charcoal stripped FBS media), they do not proliferate. We were also able to show that the c-Myb knock-down cell line forms more colonies *in vitro* and grows faster *in vivo*, data not shown by these authors. Importantly, our cell line data are highly consistent with the *in vivo* expression data of *c-Myb*, where high *c-Myb* expression occurs in slow growing, small-sized and lower grade human breast tumors, which tend to be highly responsive to hormone therapy.

**Figure 5:**
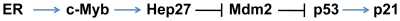
Simplified genetic schematic depicting a novel link between *c-Myb* expression and tumor suppressor p53 stabilization. Direct transcriptional targeting is depicted by blue arrows and protein-protein interactions are shown in black. The estrogen receptor directly targets c-Myb transcription and the c-Myb protein then activates Hep27 gene expression. Hep27 inhibits Mdm2, which is an inhibitor of p53, thereby stabilizing p53, leading to increased expression of p53 target gene p21 and increased cell cycle arrest.

Our results from both human breast tumor microarray data and c-Myb knock-down in MCF7 cells suggest a possible tumor suppressor role for c-Myb in breast cancer. The c-Myb proto-oncogene having tumor suppressor qualities has been observed before. Indeed, Fu *et al*., 2006 demonstrated that *c-Myb* expression inhibited fibroblast transformation *in vitro* [37]. A recent study has also found that a c-Myb target gene, *Hep27* (*DHRS2*) [25], is a novel regulator of the p53 pathway [35]. The Hep27 protein inhibits Mdm2, a well-known inhibitor of p53, thereby stabilizing the p53 protein (Fig. 5). Deisenroth *et al*. [35] observed in MCF7 cells, when *c-Myb* was exogenously expressed at increasing levels, there was a corresponding increase in both Hep27 and p53 protein levels. Conversely, when c-Myb levels were depleted via shRNA, there was a corresponding decrease in both Hep27 and p53 protein, suggesting the existence of this c-Myb-Hep27-p53 pathway in MCF7 cells.

The SAM analysis of our MCF7 c-Myb knock-down line versus parental line showed that *Hep27* expression was significantly reduced in the knock-down line (Table S2). Again, this suggests that in luminal tumors, especially Luminal A tumors where the majority are TP53 wild-type, the c-Myb-Hep27-p53 pathway may be intact and, therefore, tumors with higher levels of c-Myb will correspondingly have higher levels of a functional p53. Deisenroth *et al*. [35] analyzed breast tumor microarray data and observed higher Hep27 levels in ER+, p53 wild-type tumors, both common features of the Luminal breast tumor subtype. Luminal tumors have been repeatedly observed as chemotherapy resistant [3], [38], but until now the potential mechanism was unknown. Here, our results showing *c-Myb* high-expressing tumors having poor pathological complete response to chemotherapy (Table S1) may be due to this intact c-Myb-Hep27-p53 pathway in the Luminal subtypes. In this scenario, chemotherapeutics may elicit TP53-dependent cell cycle arrest in part via induction of p21, which has been demonstrated to occur in luminal breast tumors after neoadjuvant chemotherapy [39]. During this induced quiescence, the cells then undergo DNA repair, and upon drug removal and/or elimination, these high *c-Myb* expressing cells can re-enter the cell cycle when the DNA repair is completed. Thus, “chemoresistance” in this case may simply reflect a normal cell cycle response to DNA damage, and thus the tumor cell is responding in the same fashion as normal cells.

Our identification of a *c-Myb* gene signature was also informative from a possible developmental perspective. As compared with the Luminal tumor defining gene signature, we observed that more genes from the *c-Myb* gene list overlapped this cluster than other luminal tumor transcription factor-defining gene lists (*GATA3* and *ER*, Table S3). This is suggestive that the c-Myb transcription factor is potentially regulating many genes in luminal tumors.

During hematopoiesis the expression of *c-Myb* is highest in progenitor cell lineages and is down-regulated during differentiation [7]. We used a recent study that isolated four cell lineages in the normal mammary gland [30]: stromal, mammary stem cell, luminal progenitor, and mature luminal populations. Comparisons of our *c-Myb* gene signature, as well as the previously published *GATA3* and *ESR1* gene signatures, to the Lim *et al*. genomic data showed the opposite result for c-Myb in the mammary lineage when compared to the hematopoietic linage. Namely, the *c-Myb* gene signature (and *c-Myb* itself) significantly correlated with the mature luminal cell population, and was least expressed within the mammary stem cell enriched population (Fig. 4A and S4A). In addition, the estrogen-regulated activity was highest in both luminal progenitor and mature luminal cells, while the GATA3-regulated activity was highest in the mature luminal population, but was also increased in both the luminal progenitor and mammary stem cell populations (Figs. 4B and C).

Directly targeting c-Myb as a form of cancer therapy has been suggested and implemented in several tumor types [32], [40]. Based on the positive results, an antisense oligonucleotide targeting *c-Myb* transcript has been developed as a targeted therapeutic and a Phase I clinical trial begun for patients with advanced hematologic malignancies (National Clinical Trials Identifier: NCT00780052). It has been suggested that this form of treatment could be of value in patients with other cancers expressing high *c-Myb*, including breast tumors [7]. However, our findings indicate *c-Myb* may not be behaving as an oncogene in ER+ luminal breast tumors, which is the most common form of human breast cancer. Rather, *c-Myb* in the mammary gland is being expressed in the mature luminal cell population and acting in a pathway to stabilize the tumor suppressor, p53. Therefore, high *c-Myb* expression is beneficial in luminal breast cancer and reducing c-Myb protein levels via antisense therapy could be detrimental; as shown above, *in vitro* reduction of c-Myb via RNAi increased tumorigenesis. In total, our current findings have yielded unique insights into the role of *c-Myb* in luminal breast cancer and suggest that it may be behaving as a tumor suppressor in this disease.

## Materials and Methods

### Cell culture

#### c-Myb knock-down

MCF7 cells (a gift from F. Tamanoi, University of California-Los Angeles, Los Angeles, CA) were maintained in RPMI-1640 plus 10% FBS at 37C and 5% CO_2_. Stable knock-down of c-Myb in MCF7 cells was accomplished using a short hairpin RNA against c-Myb (shMYB; CGTTGGTCTGTTATTGCCAAGCACTTAAA) and compared to a knock-down control (shGFP) cloned into the pRS vector, purchased from OriGene (OriGene Technologies Inc., Rockville, MD; Catalog No. TR311329). Retrovial transduction was performed as described [41]. Stable populations were selected by culturing in 2 ug/mL puromycin for two weeks. Cells were then plated at clonal density and >20 colonies screened by western blotting as described [41] for c-Myb (Abcam, Cambridge, MA; ab45150), and β-tubulin (Santa Cruz Biotechnology Inc., Santa Cruz, CA; sc-9104). The clones with greatest knock-down were expanded for further analyses.

#### Addition of ZsGreen1

PT67 cells stably expressing retrovirus containing pLNCX2_ZsGreen1 (a generous gift from Dr. Kathryn B. Horwitz at the University of Colorado Health Sciences Center, Aurora, Colorado) were propagated as described [42]. MCF7 cells stably expressing shMYB or shGFP were transduced, as described above, with pLNCX2_ZsGreen1-containing retrovirus and kept under constant selection using 400 ug/mL Geneticin (Invitrogen, Carlsbad, CA; #1013127).

### *In vitro* analyses

#### Doubling Time Assay

MCF7 cells stably expressing shMYB or shGFP were seeded, in duplicate, into 10 centimeter dishes at 50,000 cells per plate. Cells were allowed 48 hours of growth prior to the first counting (t = 0), followed by counting at 48, 72, and 124 hours (Beckman Z1 Coulter Particle Counter). Doubling times were estimated by linear regression.

#### Colony Formation Assay

Soft agar assays were performed in triplicate in six-well ultra-low attachment plates (Corning Inc., Corning, NY; #3471). Briefly, a medium-agar mix was prepared by combining 2x RPMI-1640 (Invitrogen, #23400-021), 5.6 mL 1x RPMI (Invitrogen, #11875), 2.4 mL FBS (Sigma, St. Louis, MO; F6178), and 8 mL 1.8% Noble agar (Sigma, #A5431-250G). A volume of 2.3 mL of the medium-agar mix was added to each well to create a bottom layer and allowed to solidify. MCF7 cells (shMYB or shGFP) were washed with PBS, trypsinized, counted, and 8,000 cells were combined with 3 mL of medium-agar mix to create the top agar layer in each well. Once the top agar layer solidified, 0.5 mL of selective media (RPMI-1640, 10% FBS, 2 ug/mL puromycin) was added to each well and changed with fresh media every three days. Cells were grown for 15–20 days until colonies were visible. Colonies were counted by removing liquid media, adding 200 microliters of MTT dye (Promega, Madison, WI; Cell-Titer 96, #G4100), incubating for one hour at 37C, followed by scanning the plates and manual counting of colonies. Statistical significance was calculated using a two-tailed t-test.

#### MTT Assay

To estimate the IC50 of tamoxifen (Sigma-Aldrich, #T9262) on cell lines, a modified MTT assay was performed as previously described [41].

### *In vivo* tumor analysis

All animal work was approved by the University of North Carolina Institutional Animal Care and Use Committee (IACUC), protocol #07-281. MCF7 cells stably expressing shMYB+ZsGreen1 or shGFP+ZsGreen1 were collected, counted, and 550,000 cells were embedded in Matrigel and injected into each fourth mammary gland of anesthetized (2% isoflurane) Nude mice (Harlan Laboratories Inc., USA; Hsd:Athymic Nude-*Foxn1^nu^*). Tumors were allowed to grow for 15 weeks then measured by caliper. Each tumor area was calculated and statistical significance between the means of experimental versus control determined using a one-tailed independent t-test.

### Microarray analysis

All raw data from microarrays performed at the University of North Carolina are MIAME compliant and publically available at both the UNC Microarray Database [http://genome.unc.edu] as well as the Gene Expression Omnibus (GSE21371). Poly-A(+) RNA was collected (Invitrogen, Micro-FastTrack2.0 mRNA Isolation Kit) from six replicates of MCF7 cells stably expressing shGFP and five replicates expressing shMYB, reverse transcribed and labeled using the Agilent Low RNA Input Linear Amplification Kit (Agilent Technologies, Santa Clara, CA), and hybridized to Agilent Human 4×44 K Custom Oligo microarrays, adapted from manufacturer's protocol. An untreated MCF7 cell line reference was co-hybridized to each array. Microarrays were scanned on an Agilent Technologies DNA Microarray Scanner with Surescan High-Resolution Technology (Part no. G2565CA) and the image analyzed using Agilent Feature Extraction Software. Data was normalized using Lowess normalization on the Cy3 and Cy5 channels.

### Microarray statistical analyses

Supervised microarray analysis was performed on the MCF7-c-Myb RNAi vs. MCF7-vector control by selecting genes with an absolute signal intensity of at least 10 units in both dye channels and data present in at least 70% of experimental samples. A two-class, unpaired Significance Analysis of Microarrays (SAM) was performed to identify significant genes associated with *c-Myb* knock-down with a false discovery rate (FDR) of less than 1% [22].

Breast tumor microarray data on tumors without adjuvant chemotherapy (local-only Tx; NKI-147) from the Netherlands Cancer Institute [19] was used to analyze both *c-Myb*'s relation to survival, its expression across breast cancer subtypes, and its relation to both tumor size and grade. Subtypes were determined as described [3], [4], excluding the normal-like breast tumor subtype, as this subtype likely contains tumors “contaminated” with high levels of surrounding normal mammary tissue. Gene expression levels of *c-Myb* were rank ordered (high-to-low), split into halves, and relation to survival tested using the chi-square test and visualized by Kaplan-Meier survival plots (WinSTAT v.2007.1). Testing the association of *c-Myb* expression versus subtypes was performed by ANOVA using the R system for statistical computing (R Development Core Team, 2006 http://www.R-project.org). Many of these same analyses were performed on two other datasets [4], [20] (UNC, Prat *et al*. dataset: GSE18229). Normal mammary gland tissue samples (n = 13) were also analyzed in the UNC Prat *et al*. dataset.

### Gene signatures statistical analyses

The gene expression of three genes and their “activation” signatures were compared to a panel of mammary cell lineage expression data, as well as breast tumor expression data, to help elucidate their role in breast development and breast tumorigenesis. The gene signatures used in this experiment include the *c-Myb* signature described above, a *GATA3-*signature [28], and an *ER-*signature [27]; each of these three signatures were compared to the mammary cell lineage gene signatures previously defined [30]. Signatures were defined by the SAM statistics corresponding to differentially expressed genes (FDR<5%) from the model comparison, and these SAM statistics were used to weight the expression of each gene. Tumor (NKI-147 and UNC tumors) and mammary cell lineage samples [30] were evaluated by calculating the inner product of the signature gene weights and the sample. The resulting value is a relative activity measure of the signature. Boxplots were generated to compare the activity measure of each signature across tumor subtypes and sorted mammary cell lines and significance determined by ANOVA using the R system for statistical computing.

## Supporting Information

Figure S1 Gene expression of (A) *c-Myb*, (B) *ESR1* and (C) *GATA3* across the UNC tumor dataset, which includes a subset of normal mammary tissue (GSE18229; n = 324) [4]. Statistical significance was calculated by ANOVA. MaSC: mammary stem cell-enriched; LP: luminal progenitor; ML: mature luminal; St: Stromal.
(0.49 MB TIF)

Figure S2 Kaplan-Meier overall survival analysis based on *c-Myb* expression values rank ordered (high-to-low) and split into halves. NKI-147 (A) Basal-like (n = 24), (B) HER2+ (n = 29), and (C) Claudin-low (n = 10).
(0.42 MB TIF)

Figure S3 UNC breast tumor microarray dataset (Herschkowitz *et al*.; n = 232) clustered using an intrinsic gene set [3]. The luminal gene cluster, identified as genes highly correlated with the *ESR1* gene node (0.65 node correlation; 79 genes) is displayed on the right. Dendrogram branches are colored by subtype: Luminal A: dark blue, Luminal B: light blue, Basal-like: red, Normal-like: green, HER-2 enriched: pink.
(5.41 MB TIF)

Figure S4 Gene expression of (A) *c-Myb*, (B) *ESR1* and (C) *GATA3* across NKI-147 and mammary gland cell lineage gene signatures [29]. Statistical significance was calculated by ANOVA. MaSC: mammary stem cell-enriched; LP: luminal progenitor; ML: mature luminal; St: Stromal.
(0.75 MB TIF)

Figure S5 (A) *c-Myb*, (B) *ESR1*, and (C) *GATA3* regulated activities across a UNC breast tumor dataset which includes a subset of normal mammary gland tissue (GSE18229; n = 324, [4]) or mammary gland cell lineage gene signatures. Tumor gene expression was weighted according to *ESR1*, *GATA3*, or *c-Myb* gene signatures by calculating the inner product of each signature and the tumor expression profile. Lim *et al*. lineage signatures were processed in the same fashion as tumors to demonstrate the developmental context of these signatures. MaSC: mammary stem cell-enriched; LP: luminal progenitor; ML: mature luminal; St: Stromal.
(0.70 MB TIF)

Table S1 *c-Myb* high expression correlates with low pCR. Pathologic complete response (pCR) data of Hess *et al*., (2006) rank ordered (high-to-low), split into halves based on *c-Myb* expression values, and analyzed by chi-square.
(0.27 MB PDF)

Table S2 *c-Myb* gene signature identified by knock-down of endogenous c-Myb in MCF7 cells. A supervised analysis was performed using SAM with five replicates of MCF7 cells stably expressing shMYB versus six replicates of the control line expressing shGFP. A list of 2,088 genes (892 positive; 1196 negative) with an FDR<1% was obtained.
(0.16 MB XLSX)

Table S3 *c-Myb*, *ESR1*, and *GATA3* gene signatures define many luminal cluster genes. GenBank accession numbers and gene symbols of the 79 genes highly correlated with the *ESR1* gene node (0.65 node correlation). Overlapping genes with *c-Myb*, *ESR1*, and *GATA3* gene signatures are listed.
(0.01 MB XLSX)
