# Supplementary figures and images for: Potential Tumor Suppressor Role for the c-Myb Oncogene in Luminal Breast Cancer
Source: PLoS One. 2010 Oct 7;5(10):e13073. doi: 10.1371/journal.pone.0013073 (PMC2951337; doi:10.1371/journal.pone.0013073)

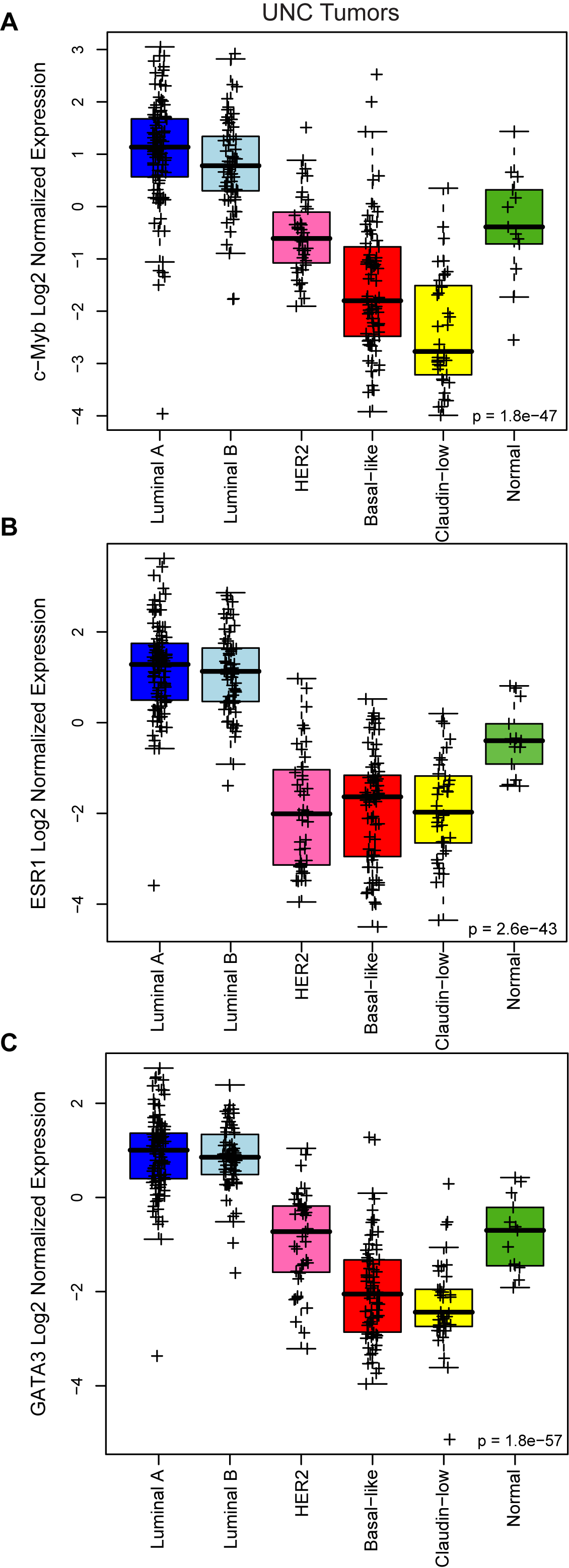

Supplement: Figure S1 — Gene expression of (A) c-Myb, (B) ESR1 and (C) GATA3 across the UNC tumor dataset, which includes a subset of normal mammary tissue (GSE18229; n = 324) [4]. Statistical significance was calculated by ANOVA. MaSC: mammary stem cell-enriched; LP: luminal progenitor; ML: mature luminal; St: Stromal. (0.49 MB TIF) [file pone.0013073.s001.tif]

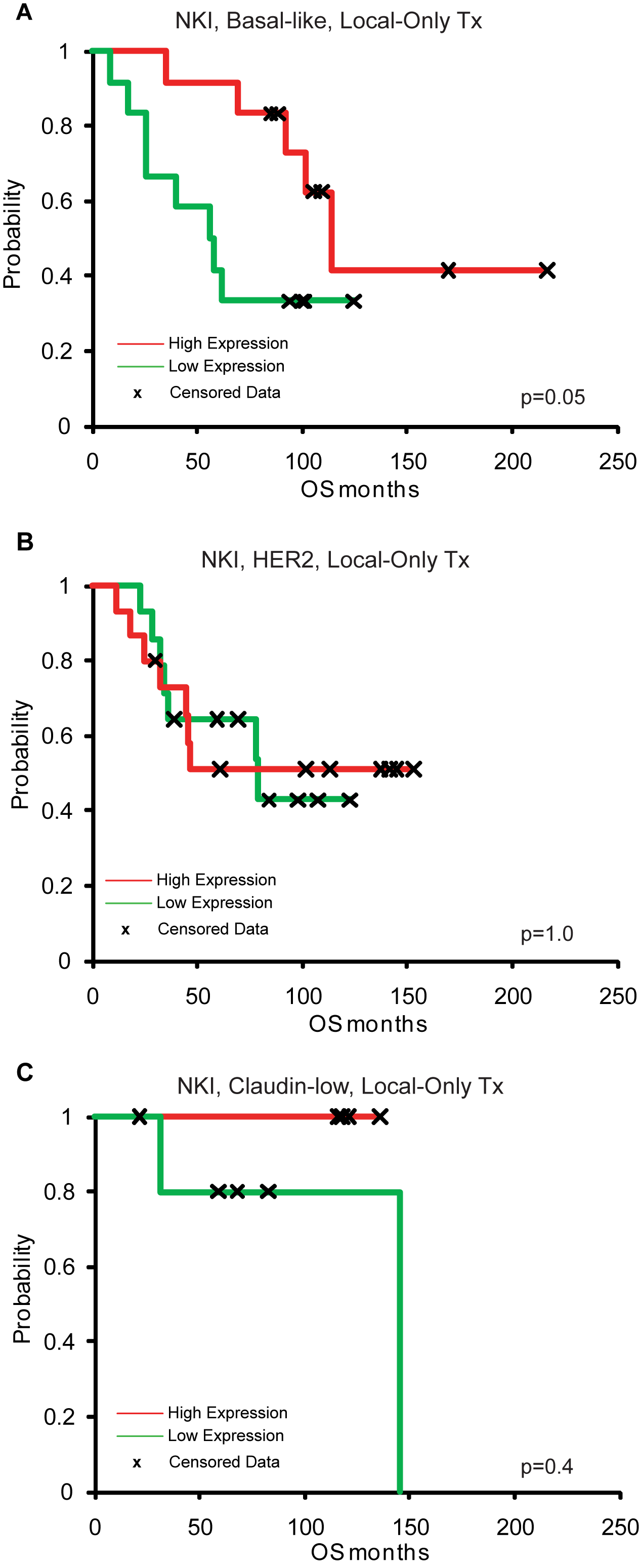

Supplement: Figure S2 — Kaplan-Meier overall survival analysis based on c-Myb expression values rank ordered (high-to-low) and split into halves. NKI-147 (A) Basal-like (n = 24), (B) HER2+ (n = 29), and (C) Claudin-low (n = 10). (0.42 MB TIF) [file pone.0013073.s002.tif]

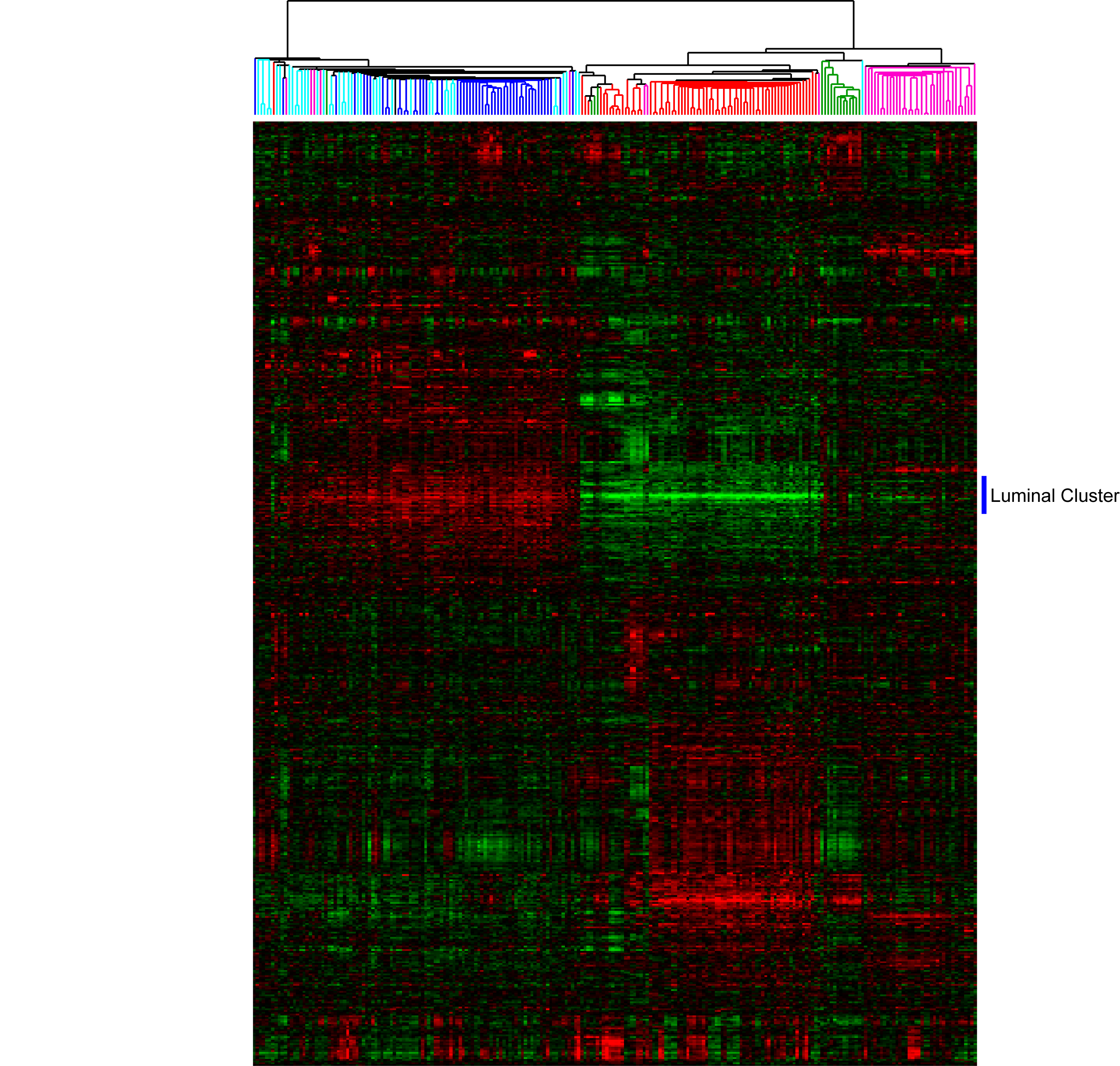

Supplement: Figure S3 — UNC breast tumor microarray dataset (Herschkowitz et al.; n = 232) clustered using an intrinsic gene set [3]. The luminal gene cluster, identified as genes highly correlated with the ESR1 gene node (0.65 node correlation; 79 genes) is displayed on the right. Dendrogram branches are colored by subtype: Luminal A: dark blue, Luminal B: light blue, Basal-like: red, Normal-like: green, HER-2 enriched: pink. (5.41 MB TIF) [file pone.0013073.s003.tif]

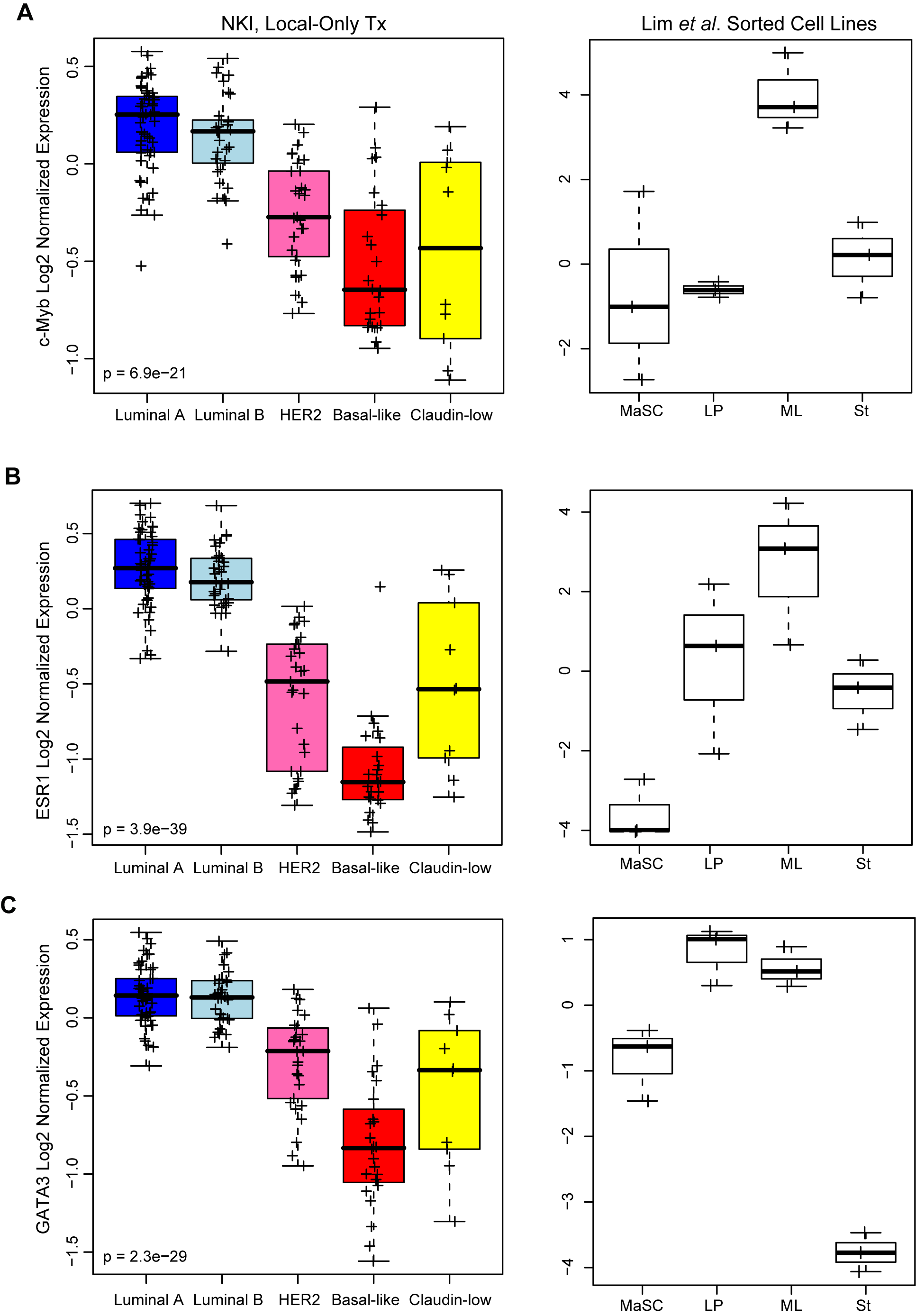

Supplement: Figure S4 — Gene expression of (A) c-Myb, (B) ESR1 and (C) GATA3 across NKI-147 and mammary gland cell lineage gene signatures [29]. Statistical significance was calculated by ANOVA. MaSC: mammary stem cell-enriched; LP: luminal progenitor; ML: mature luminal; St: Stromal. (0.75 MB TIF) [file pone.0013073.s004.tif]

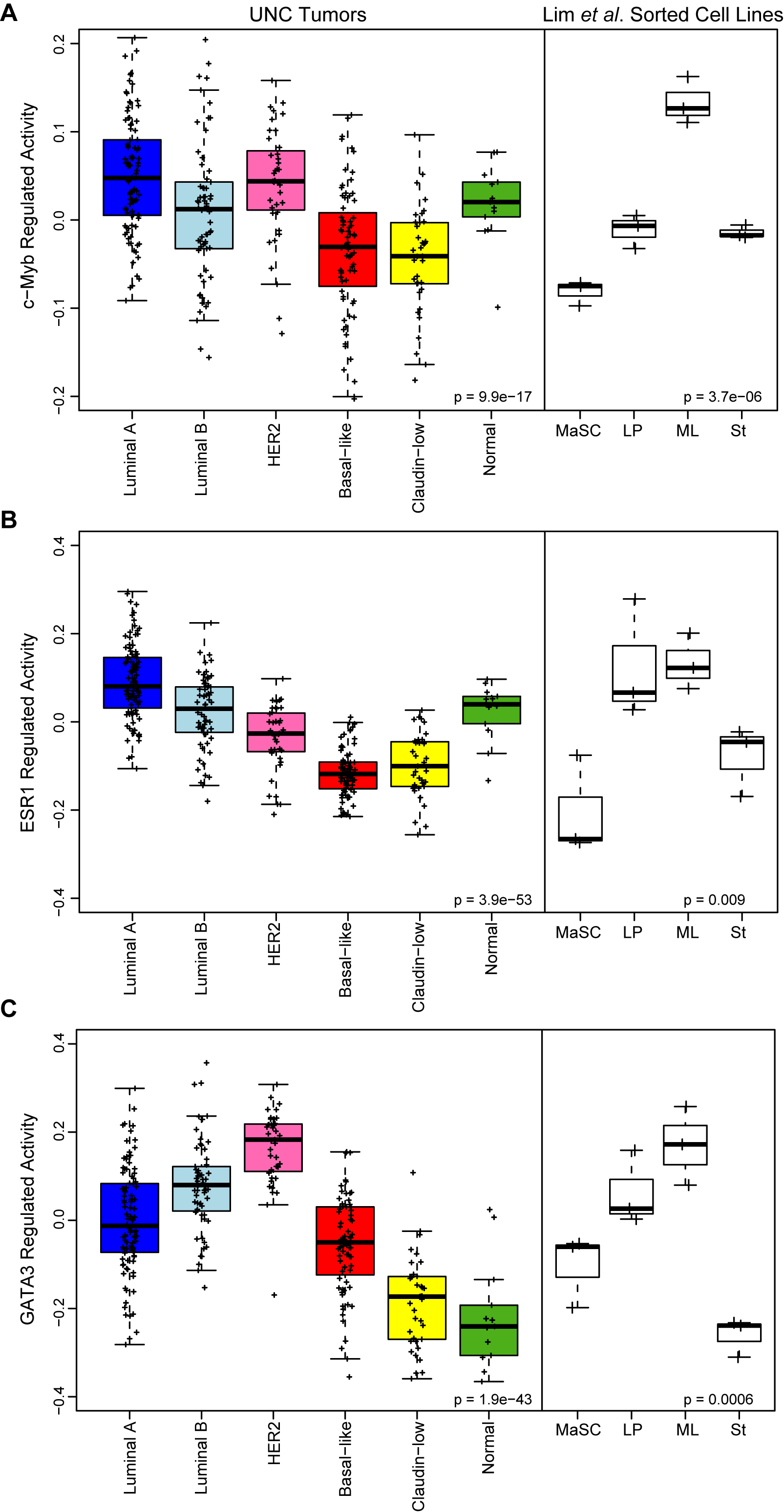

Supplement: Figure S5 — (A) c-Myb, (B) ESR1, and (C) GATA3 regulated activities across a UNC breast tumor dataset which includes a subset of normal mammary gland tissue (GSE18229; n = 324, [4]) or mammary gland cell lineage gene signatures. Tumor gene expression was weighted according to ESR1, GATA3, or c-Myb gene signatures by calculating the inner product of each signature and the tumor expression profile. Lim et al. lineage signatures were processed in the same fashion as tumors to demonstrate the developmental context of these signatures. MaSC: mammary stem cell-enriched; LP: luminal progenitor; ML: mature luminal; St: Stromal. (0.70 MB TIF) [file pone.0013073.s005.tif]
