# Supplementary material for: Potential Tumor Suppressor Role for the c-Myb Oncogene in Luminal Breast Cancer
Source: PLoS One. 2010 Oct 7;5(10):e13073. doi: 10.1371/journal.pone.0013073 (PMC2951337; doi:10.1371/journal.pone.0013073)
Supplement: Table S1 — c-Myb high expression correlates with low pCR. Pathologic complete response (pCR) data of Hess et al., (2006) rank ordered (high-to-low), split into halves based on c-Myb expression values, and analyzed by chi-square. (0.27 MB PDF) [file pone.0013073.s006.pdf]

Table S1. c-Myb high expression correlates with poor pCR

c-Myb:	High	Low
No pCR	55	44
pCR	11	23

χ^2=4.6, df=1, p=0.03
